# Supplementary material for: Hybrid Models and Biological Model Reduction with PyDSTool
Source: PLoS Comput Biol. 2012 Aug 9;8(8):e1002628. doi: 10.1371/journal.pcbi.1002628 (PMC3415397; doi:10.1371/journal.pcbi.1002628)
Supplement: Text S4 — Complete source code for the PyDSTool package (version 0.88.120504). Includes API documentation and help files linking to web pages. This file is identical to the current public release on Sourceforge.net. (ZIP) [file pcbi.1002628.s004.zip › PyDSTool/html/PyDSTool.Toolbox.dssrt-module.html]

xml version="1.0" encoding="ascii"?


PyDSTool.Toolbox.dssrt


| Home | Trees | Indices | Help | PyDSTool |
| --- | --- | --- | --- | --- |

Package PyDSTool ::
Package Toolbox ::
Module dssrt

[hide private]

[frames] | no frames]

# Module dssrt

source code

Implementation of dominant scale analysis techniques for python.

Loosely based on Matlab tool DSSRT. This module is incomplete and is
still a work in progress! See my publications on this subject for further
details.

Robert Clewley, 2009


|  |  |
| --- | --- |
| Classes | [hide private] |

join\_actives

leave\_actives

become\_most\_dominant

join\_fast

join\_slow

leave\_fast

leave\_slow

is\_active

is\_inactive

is\_modulatory

is\_most\_dominant

is\_fast

is\_slow

is\_order1

regime

epoch

domscales

dssrt\_assistant

Scorer

EpochSeqScorer

VarAlphabet


|  |  |
| --- | --- |
| Functions | [hide private] |

|  |  |
| --- | --- |
| find\_ep\_ix(eps, t)  Finds epoch during time t | source code |

|  |  |
| --- | --- |
| find\_regime\_transition(criteria\_list, min\_t=-inf) | source code |

|  |  |
| --- | --- |
| \_comp(a, b, eps, atol) | source code |

|  |  |
| --- | --- |
| transition\_psi(epoch, pt, atol)  Assume that, generically, there will only be one transition at a time. | source code |

|  |  |
| --- | --- |
| transition\_tau(epoch, pt, atol)  Assume that, generically, there will only be one transition at a time. | source code |

|  |  |
| --- | --- |
| check\_opts(opts) | source code |

|  |  |
| --- | --- |
| define\_psi\_events(acts\_all, mods\_all, focus\_var, ignore\_transitions=None) | source code |

|  |  |
| --- | --- |
| define\_tau\_events(slow, fast, order1, ref) | source code |

|  |  |
| --- | --- |
| get\_taus(pts, tau\_names)  pts can be a Point or Pointset | source code |

|  |  |
| --- | --- |
| get\_infs(pts, inf\_names)  pts can be a Point or Pointset | source code |

|  |  |
| --- | --- |
| split\_pts(pts, interval, reset\_t0=None)  Return trajectory made up from pts argument restricted to the given interval. | source code |

|  |  |
| --- | --- |
| find\_epoch\_period(epochs, verbose=False) | source code |

|  |  |
| --- | --- |
| split(ds\_pt, thresh)  Split this grouping of values into sub-groups according to spectral gaps at threshold thresh | source code |

|  |  |
| --- | --- |
| as\_absolute\_ratios(vals)  Express a single time point's values as ratios of the largest (>1). | source code |

|  |  |
| --- | --- |
| as\_relative\_ratios(vals)  Express a single time point's relative values as successive ratios (>1). | source code |

|  |  |
| --- | --- |
| spectral\_gaps\_relative(as\_rel\_ratios, thresh)  List of indices of spectral gaps larger than thresh in size | source code |

|  |  |
| --- | --- |
| spectral\_gaps\_absolute(as\_abs\_ratios, thresh)  List of indices of spectral gaps larger than thresh in size | source code |

|  |  |
| --- | --- |
| partition\_range(range\_tuple, split\_ixs) | source code |

|  |  |
| --- | --- |
| normalized\_psis(epochs, root, midpoint\_only=True, small=1e-16)  Returns Pointset of normalized\* psis based on contents of 'epochs' argument, which would be returned from running da.domscales['psi'].calc\_epochs() where da is a domscales assistant class. | source code |

|  |  |
| --- | --- |
| show\_epochs(eps)  Small utility to print more detailed information about epochs | source code |

|  |  |
| --- | --- |
| plot\_psis(da, cols=None, do\_vars=None, do\_log=True, use\_prefix=True)  da is a dssrt\_assistant object. | source code |

|  |  |
| --- | --- |
| get\_symbol\_sequence(epoch\_list, get\_actives=True, get\_modulatory=False) | source code |

|  |  |
| --- | --- |
| strip\_speed(seq) | source code |

|  |  |
| --- | --- |
| pad(seq, n) | source code |

|  |  |
| --- | --- |
| only\_dynamic(seq, dynamic\_vars) | source code |

|  |  |
| --- | --- |
| comp\_seqs(seq1, seq2, scorer)  Based on code by Gergely Szollosi | source code |

|  |  |
| --- | --- |
| swdist(str1, str2, scorer, common\_divisor=`'``longest``'`, min\_threshold=None)  Return approximate string comparator measure (between 0.0 and 1.0) using the Smith-Waterman distance. | source code |

|  |  |
| --- | --- |
| editdist\_edits(str1, str2)  Return approximate string comparator measure (between 0.0 and 1.0) using the edit (or Levenshtein) distance as well as a triplet with the counts of the actual edits (inserts, deletes and substitutions). | source code |

|  |  |
| --- | --- |
| jaro(str1, str2, min\_threshold=None)  Return approximate string comparator measure (between 0.0 and 1.0) | source code |

|  |  |
| --- | --- |
| tabulate\_epoch\_seqs(epseq1, epseq2)  Create a text table of epochs | source code |

|  |  |
| --- | --- |
| indent(rows, hasHeader=False, headerChar=`'``-``'`, delim=`'` `\|` `'`, justify=`'``left``'`, separateRows=False, prefix=`'``'`, postfix=`'``'`, wrapfunc=<function <lambda> at 0x96bea30>)  Indents a table by column. | source code |


|  |  |
| --- | --- |
| Variables | [hide private] |

\_classes = `['epoch', 'regime', 'dssrt_assistant', 'domscales',...`

\_predicates = `['is_active', 'is_inactive', 'is_modulatory', 'i...`

\_functions = `['find_epoch_period', 'transition_psi', 'transiti...`

MATCH = `'match'`

MISMATCH = `'mismatch'`

CLOSE = `'close'`

FAR = `'far'`

VCLOSE = `'vclose'`

VFAR = `'vfar'`

GAP = `'gap'`

APPROX = `'approx'`

JARO\_MARKER\_CHAR = `'*'`


|  |  |
| --- | --- |
| Function Details | [hide private] |

|  |  |
| --- | --- |
| split\_pts(pts, interval, reset\_t0=None) | source code |

Return trajectory made up from pts argument restricted to the given
interval.

Use reset\_t0 = T (float) to reset output trajectory t0 to T. Default
is to use native time frame of pts argument.

|  |  |
| --- | --- |
| as\_absolute\_ratios(vals) | source code |

Express a single time point's values as ratios of the largest (>1).
first entry is ratio of largest to itself == 1.

|  |  |
| --- | --- |
| as\_relative\_ratios(vals) | source code |

Express a single time point's relative values as successive ratios
(>1). first entry is ratio of largest to itself == 1.

|  |  |
| --- | --- |
| normalized\_psis(epochs, root, midpoint\_only=True, small=1e-16) | source code |

```
Returns Pointset of normalized* psis based on contents of 'epochs' argument,
which would be returned from running da.domscales['psi'].calc_epochs()
where da is a domscales assistant class.

  * Largest influence always normalized to 1 for every time point.
```

|  |  |
| --- | --- |
| plot\_psis(da, cols=None, do\_vars=None, do\_log=True, use\_prefix=True) | source code |

da is a dssrt\_assistant object. cols is an optional dictionary mapping
names of Psi entries to specific color/style character codes. Pass
do\_vars list of Psi names to restrict, otherwise all will be plotted.
Option to plot on vertical log scale.

Option to switch off 'psi\_' + da's focus\_var as prefix for
coordnames.

Requires matplotlib.

|  |  |
| --- | --- |
| swdist(str1, str2, scorer, common\_divisor=`'``longest``'`, min\_threshold=None) | source code |

```
Return approximate string comparator measure (between 0.0 and 1.0)
   using the Smith-Waterman distance.

USAGE:
  score = swdist(str1, str2, scorer, common_divisor, min_threshold)

ARGUMENTS:
  str1            The first sequence
  str2            The second sequence
  scorer          scorer object instance to compare two symbols
  common_divisor  Method of how to calculate the divisor, it can be set to
                  'average','shortest', or 'longest' , and is calculated
                  according to the lengths of the two input strings
  min_threshold   Minimum threshold between 0 and 1

DESCRIPTION:
  Smith-Waterman distance is commonly used in biological sequence alignment.

  Scores for matches, misses, gap and extension penalties are set to values
  described in the scorer
```

|  |  |
| --- | --- |
| editdist\_edits(str1, str2) | source code |

```
Return approximate string comparator measure (between 0.0 and 1.0)
   using the edit (or Levenshtein) distance as well as a triplet with the
   counts of the actual edits (inserts, deletes and substitutions).

USAGE:
  score, edit_counts = editdist_edits(str1, str2)

ARGUMENTS:
  str1           The first seq
  str2           The second seq

DESCRIPTION:
  The edit distance is the minimal number of insertions, deletions and
  substitutions needed to make two strings equal.

  edit_counts  is a list with three elements that contain the number of
               inserts, deletes and substitutions that were needed to convert
               str1 into str2.

  For more information on the modified Soundex see:
  - http://www.nist.gov/dads/HTML/editdistance.html
```

|  |  |
| --- | --- |
| jaro(str1, str2, min\_threshold=None) | source code |

```
Return approximate string comparator measure (between 0.0 and 1.0)

USAGE:
  score = jaro(str1, str2, min_threshold)

ARGUMENTS:
  str1           The first string
  str2           The second string
  min_threshold  Minimum threshold between 0 and 1 (currently not used)

DESCRIPTION:
  As desribed in 'An Application of the Fellegi-Sunter Model of
  Record Linkage to the 1990 U.S. Decennial Census' by William E. Winkler
  and Yves Thibaudeau.
```

|  |  |
| --- | --- |
| indent(rows, hasHeader=False, headerChar=`'``-``'`, delim=`'` `\|` `'`, justify=`'``left``'`, separateRows=False, prefix=`'``'`, postfix=`'``'`, wrapfunc=<function <lambda> at 0x96bea30>) | source code |

Indents a table by column.

- rows: A sequence of sequences of items, one sequence per row.
- hasHeader: True if the first row consists of the columns' names.
- headerChar: Character to be used for the row separator line (if
  hasHeader==True or separateRows==True).
- delim: The column delimiter.
- justify: Determines how are data justified in their column. Valid
  values are 'left','right' and 'center'.
- separateRows: True if rows are to be separated by a line of
  'headerChar's.
- prefix: A string prepended to each printed row.
- postfix: A string appended to each printed row.
- wrapfunc: A function f(text) for wrapping text; each element in the
  table is first wrapped by this function.

  


|  |  |
| --- | --- |
| Variables Details | [hide private] |

### \_classes

Value:
:   ```
    ['epoch',
     'regime',
     'dssrt_assistant',
     'domscales',
     'Scorer',
     'EpochSeqScorer',
     'VarAlphabet']
    ```

### \_predicates

Value:
:   ```
    ['is_active',
     'is_inactive',
     'is_modulatory',
     'is_most_dominant',
     'is_fast',
     'is_slow',
     'is_order1',
     'join_actives',
    ...
    ```

### \_functions

Value:
:   ```
    ['find_epoch_period',
     'transition_psi',
     'transition_tau',
     'get_infs',
     'get_taus',
     'split_pts',
     'define_psi_events',
     'normalized_psis',
    ...
    ```

  


| Home | Trees | Indices | Help | PyDSTool |
| --- | --- | --- | --- | --- |

|  |  |
| --- | --- |
| Generated by Epydoc 3.0.1 on Fri May 4 15:24:04 2012 | http://epydoc.sourceforge.net |
